# Society Notes

**Published:** 1905-02

**Authors:** 


### Society Notes.

---

THE FIFTH DISTRICT MEDICAL SOCIETY OF TEXAS held its third semi-annual meeting in San Antonio, Texas, Tuesday and Wednesday, February 7th and 8th. Place of meeting, Elks' Club Rooms.

The first session of the meeting was held at 3 p. m., February 7th. Then there was a meeting of the House of Delegates, for the transaction of such business as might be deemed necessary. The second session was held at 8 p. m., when the following committees reported through their chairmen. These reports were very exhaustive and instructive, and the society was so much impressed by them that a motion was made and carried that these committees be made permanent:

Report of Committee on Proprietary and Synthetic Preparations, Dr. John V. Spring, Chairman, San Antonio; report of Committee on Pure Foods and Drugs, Dr. W. E. Luter, Chairman, San Antonio; report of Committee on Social Evils, Dr. Moody, Chairman, San Antonio; report of Committee on Quarantine and Health Laws, Dr. Frank Paschal, Chairman, San Antonio; report of Committee on Patent Medicines and the Practice of Drugless Doctors, Dr. B. F. Kingsley, Chairman, San Antonio.

At the morning session, February 8th, the following very able papers were read and discussed:

"Modern Therapeutics and Pharmacy,"* Dr. F. Hadra, San Antonio; "Snakebite and Its Treatment," Dr. J. T. FitzSimon, Castroville. At the afternoon session Dr. D. Y. Wilbern, of Runge, Texas, read a paper on "The Treatment of Typhoid Fever." Dr. C. H. Wilkinson, of San Antonio, read a paper entitled, "Should Man Be Restricted in His Indiscriminate Use of Alcohol? If so, How?" "Anchylostomiasis—Report of a Case," Dr. B. F. Stout, San Antonio; "Treatment of Strictures of the Urethra," Dr. F. Paschal, San Antonio.

Much interest was manifested throughout the entire session, and the papers very ably discussed. At the evening session the report of the Legislative Committee was read by Dr. F. Paschal, Chairman. The report was approved, and the committee urged to continue its fight upon the proposed Osteopathic bill and favor legislation for the suppression of quacks and drugless doctors.

The time having arrived for the election of officers, the following officers were elected: President, Dr. M. B. Grace, Seguin; Secretary, Dr. W. A. King, Falls City; Treasurer, Dr. J. T. FitzSimon, Castroville. The event of this session, however, was the masterly address of the retiring President, Dr. Malone Duggan, of Eagle Pass, Texas. After which the society adjourned to the banquet hall, thus closing one of the most instructive and interesting meetinges this society ever held.

---

*This paper will appear in the March number of the JOURNAL.

Kaufman County Medical Society.—Terrell, February 14th inst. B. J. Hubbard, President; W. J. Pollard, Secretary.

Brazos Valley Medical Society.—Cameron, Texas, May 9th and 10th. E. N. Shaw, President; W. B. Briggs, Secretary.

The Central Texas Medical Society held its regular meeting at Waco, January 10th and 11th. No report received. J. W. McCutchan, President; W. R. Thompson, Fort Worth, Secretary and Treasurer.

Hunt County Medical Society elected officers for 1905 as follows: G. F. Floyd, Lone Oak, President; C. T. Kennedy, Greenville, Vice-President; M. L. Moody, Greenville, Secretary. Delegates to State Association, Moody, Coppege, Norris.

